# Associations between Lifestyle Factors and Neurocognitive Impairment among Chinese Adolescent and Young Adult (AYA) Survivors of Sarcoma

**DOI:** 10.3390/cancers15030799

**Published:** 2023-01-28

**Authors:** Yin Ting Cheung, Chung Tin Ma, Michael Can Heng Li, Keary Rui Zhou, Herbert Ho Fung Loong, Agnes Sui Yin Chan, Kwok Chuen Wong, Chi Kong Li

**Affiliations:** 1 School of Pharmacy, Faculty of Medicine, The Chinese University of Hong Kong, Hong Kong, China; 2 Department of Clinical Oncology, Faculty of Medicine, The Chinese University of Hong Kong, Hong Kong, China; 3 Department of Psychology, The Chinese University of Hong Kong, Hong Kong, China; 4 Department of Orthopaedics & Traumatology, Prince of Wales Hospital, Hong Kong, China; 5 Department of Paediatrics, Faculty of Medicine, The Chinese University of Hong Kong, Hong Kong, China; 6 Department of Paediatrics & Adolescent Medicine, Hong Kong Children’s Hospital, Hong Kong, China; 7 Hub of Paediatric Excellence, The Chinese University of Hong Kong, Hong Kong, China

**Keywords:** young-adult cancer, pediatric cancer, sarcoma, cancer survivors, neurocognitive impairment, lifestyle, adolescent and young adult

## Abstract

**Simple Summary:**

Young survivors of bone tumors and soft-tissue sarcoma are at risk of cognitive impairment due to their previous cancer therapies and the development of cancer-/treatment-related chronic health conditions as they age. We postulate that a compromised health status, coupled with the continued engagement in an unhealthy lifestyle during survivorship, may further exacerbate cognitive impairment among survivors. Our study found that survivors of bone tumors and soft-tissue sarcoma demonstrated impairment in attention, processing speed and executive function (higher order thinking ability). Low physical activity and smoking were associated with inattention. Survivors who worked >9 h per day had worse executive function than those with shorter working hours. Of note is that survivors who had a chronic health condition and were physically inactive demonstrated the worst attention and executive function performance. Our findings support continual efforts to investigate intervention targets and leverage health behaviors as modifiable risk factors to prevent cognitive dysfunction in this population.

**Abstract:**

Background: The effect of lifestyle on neurocognitive impairment among cancer survivors remain an understudied area. This study explored the association between lifestyle factors and neurocognitive outcomes (specifically, attention, memory, processing speed and cognitive flexibility) in AYA survivors (aged 15–39 years) of sarcoma. Methods: This study recruited 116 AYA survivors (age 28.2 (SD = 8.2) years), who were diagnosed with osteosarcoma (49%) or soft-tissue sarcoma (51%) at age 13.3 (SD = 7.2) years. The neurocognitive battery included measures of attention, memory, motor-processing speed, and cognitive flexibility. Survivors reported health-damaging practices, which included: physical inactivity, smoking, alcohol intake, inadequate sleep (<7 h of actual sleep/day), sleep-related fatigue (Multidimensional Fatigue Scale) and long working hours (>9 h/day). General linear modeling was conducted to examine the association between lifestyle factors and neurocognitive outcomes, adjusting for age at diagnosis, sex, education attainment and clinical/treatment variables. Results: At 14.9 (SD = 7.6) years post-diagnosis, survivors demonstrated impairment in attentiveness (4.3–13.0%), processing speed (34.5%) and cognitive flexibility (18.1%). Nearly half (45.7%) had developed a chronic health condition (CHC). Low physical activity (estimate = −0.97, *p* = 0.003) and sleep-related fatigue (estimate = −0.08, *p* = 0.005) were associated with inattention. Survivors who worked >9 h/day (n = 15) demonstrated worse attention (estimate = 5.42, *p* = 0.023) and cognitive flexibility (estimate = 5.22, *p* = 0.005) than survivors who worked ≤9 h/day (n = 66). Interaction analysis (CHCs*physical activity) showed that survivors who developed CHCs and reported low physical activity had worse attention (*p* = 0.032) and cognitive-flexibility (*p* = 0.019) scores than other subgroups. Conclusion: Treatment-related CHCs, coupled with continued physical inactivity, may exacerbate inattention and executive dysfunction among survivors. Long working hours and sleep-related fatigue are associated with worse functioning; this finding should be validated with prospective assessment of work-related stressors and objective sleep measures.

## 1. Introduction

Osteosarcoma and soft-tissue sarcomas (STS) are relatively rare in the pediatric and young-adult populations compared with malignancies such as leukemia, lymphoma, brain tumors and thyroid cancers [1]. In Hong Kong, there were seven newly diagnosed cases of osteosarcoma and six newly diagnosed cases of STS every year between 2015 and 2019 in the pediatric (≤15 years old) population [2]. Among young adults (>15 to 39 years old), there was an average of 12 new cases of osteosarcoma and 40 new cases of STS diagnosed each year during the same period [2]. Improved treatment strategies for osteosarcoma and STS have led to increased survival rates and an emerging population of adolescent and young-adult (AYA) survivors of sarcoma [1]. However, survivorship comes at the cost of developing a myriad of treatment-related chronic health conditions (CHCs) and persistent effects that can affect the survivor’s functional outcomes later in life [3,4].

One study in the US conducted performance-based neurocognitive assessment in 117 survivors of osteosarcoma (median age 37.6 years) and 90 survivors of Ewing sarcoma (median age 34.2 years); they reported that nearly a quarter of the survivors demonstrated moderate-to-severe impairments in measures of executive function (11.6–23.8%), attention (10.5–7.9%) and memory (9.0–11.4%) [5]. Compared with survivors of acute lymphoblastic leukemia (ALL) and brain tumors, few studies have explored the neurocognitive sequelae of treatment among survivors of sarcoma [3,6,7,8] and none of these were conducted in Chinese populations. Within the Asian population, one Singaporean study (n = 79, 86% Chinese patients in the study cohort) found that 17.8% of the sarcoma patients reported “difficulty concentrating” [9]. Though the literature in this area remains scarce, these studies suggested that survivors of sarcoma might experience long-term cognitive deficits which can affect their functional outcomes. Of note is that neurocognitive function is an important outcome among AYA survivors because adolescence and young adulthood mark a crucial stage in their psychosocial development and fostering functional independence. For example, survivors of osteosarcoma and Ewing sarcoma who demonstrated neurocognitive impairment were significantly associated with lower rates of college-degree attainment, lower rates of full-time employment and lower personal income [3,6,7,8].

There is extensive evidence demonstrating the impact of central nervous system (CNS)-directed therapies and exposure to high-dose methotrexate (HDMTX) on neurocognitive function among survivors of childhood ALL [10]. One would expect osteosarcoma survivors to be more vulnerable to the neurotoxic effects of HDMTX, as they typically receive four to five times higher HDMTX exposure than ALL survivors treated with contemporary chemotherapy-only protocols [11,12]. However, studies on sarcoma cohorts have reported that neurocognitive impairment in osteosarcoma and STS survivors may be attributable to comorbid conditions resulting from cancer treatment rather than the direct neuroeffects of drugs used for treatment [3,7,8]. Drugs that are not CNS-directed can also induce neurotoxicity through other treatment-related CHCs. For example, anthracyclines-related cardiopulmonary late effects might induce chronic vascular injury and inflammation, which may contribute to cerebrovascular pathology and manifest as neurocognitive impairment [13]. There is still inconclusive evidence on whether adverse functional outcomes in survivors of sarcoma may be attributable to these treatment-related CHCs.

Disease- and treatment-related factors only partially account for functional outcomes. Emerging evidence shows that lifestyle is an important factor that contributes to neurodevelopmental processes among young survivors [14]. Examining health behaviors in AYAs is particularly relevant because they are passing through a unique phase of life in which they develop lifelong habits and may have significant implications on their health and psychosocial outcomes. We reported, in a previous study, that up to two-thirds of the survivors of childhood cancer in Hong Kong are not engaging in the recommended health-protective practices [15]. The increased risk of developing treatment-related CHCs coupled with continued engagement in an unhealthy lifestyle during survivorship may exacerbate neurocognitive impairment among survivors. Lifestyle factors have been found to be linked to neurocognitive function in the general population, but their effects on neurocognitive function among AYA survivors of cancer remain an understudied area [14]. A recent study demonstrated an association between exercise intolerance and neurocognitive impairment among survivors of childhood ALL [16]. Another study reported the detrimental effects of alcohol intake on neurocognitive function among adult survivors of childhood cancer [17]. No study has investigated whether lifestyle factors affect neurocognitive outcomes in young survivors of sarcoma in Hong Kong, where a sedentary lifestyle, hectic work culture and urban environmental stressors are prevalent in the AYA population.

The objectives of this study were to explore the associations between lifestyle factors and neurocognitive outcomes (specifically, attention, memory, processing speed and cognitive flexibility) in a cohort of Chinese AYA survivors of osteosarcoma and STS. We also investigated potential interactions between lifestyle factors and CHCs, and their effects on these neurocognitive outcomes.

## 2. Methods

### 2.1. Study Design

This cross-sectional study was conducted between June 2020 and May 2022 at the long-term follow-up (LTFU) clinics of the Department of Orthopaedics and Traumatology and the Department of Paediatrics of the Prince of Wales Hospital in Hong Kong. The research procedure included a prospective neurocognitive assessment and survey, and a retrospective review of the participants’ previous cancer treatment/clinical data from the Clinical Management System (CMS), an in-house electronic health records. This study was approved by the Joint Chinese University of Hong Kong–New Territories East Cluster Clinical Research Ethics Committee (Ref: 2018.131). The participants who were ≥18 years old provided written informed consent, while the participants who were <18 years provided written assent and written consent by their legal guardians.

### 2.2. Study Population

Eligible participants were recruited through convenience sampling. Investigators obtained the list of patients who were due for follow-up consultation at the LTFU clinics, which typically occurred once every week. Patients were then screened for eligibility using the CMS. All eligible patients who subsequently attended the LTFU clinic were invited to participate in the study.

Survivors were eligible for the study if they (1) were aged 15 to 39 years old at the time of recruitment, (2) were diagnosed with osteosarcoma or STS, and (3) had survived at least 2 years post-cancer treatment. Survivors who had any pre-existing condition associated with cognitive impairment (e.g., autism, severe head injuries) were excluded.

### 2.3. Treatment Regimens

Osteosarcoma survivors were treated according to the Hong Kong Paediatric Haematology and Oncology Study Group 1993 osteosarcoma regimen [18,19], which was adapted from well-established international protocols [11,12]. It included 8 weeks of neoadjuvant chemotherapy with cisplatin, doxorubicin and HDMTX followed by tumor resection at week 10 and post-operative chemotherapy [18]. The treatment duration was 27 weeks among good responders with ≥90% tumor necrosis, or 40 weeks with methotrexate replaced by ifosfamide and etoposide among poor responders. Young adults (>18 years) of osteosarcoma also typically received surgery and chemotherapy (HDMTX, doxorubicin, cisplatin, and/or etoposide). Metastasectomy, whenever surgically feasible, was conducted for survivors with confirmed lung metastasis or patients with cancer recurrence in the lungs.

STS survivors received heterogeneous adjuvant or neoadjuvant protocols, which typically included a combination of doxorubicin, vincristine, ifosfamide, actinomycin D and cyclophosphamide.

### 2.4. Study Outcomes

At follow-up, survivors completed a performance-based neurocognitive battery with trained research assistants under the supervision of the investigators. The cognitive domains of interest are similar to those investigated in other cognitive studies on survivors of childhood cancer [3,13,20], and included the Continuous Performance Test [CPT]-III) for attention [21], the Modified Taylor Complex Figure for memory [22], the Trail Making Test Part A and Grooved Pegboard for processing speed [23], and the Trail Making Test Part B for cognitive flexibility [23]. The neurocognitive outcomes were transformed into age-adjusted t-scores (mean = 50, standard deviation (SD) = 10) using the normative data provided by the test manuals or the published literature [24,25,26] (Supplement Table S1).

### 2.5. Lifestyle Factors

The survivors completed a 20-min structured questionnaire to report their lifestyle habits. The predictors of interest were lifestyle factors most frequently reported in studies of childhood cancer survivors in other countries and that demonstrated associations with neurocognitive impairment in the cancer or general populations. They are low physical activity [16,27,28], substance use (alcohol, smoking and illicit drugs) [17,29,30,31], inadequate sleep [32,33,34], and long working hours [35].

(1) *Low physical activity.* The 11-point scale Chinese University of Hong Kong: Physical Activity Rating for Children and Youth (CUHK-PARCY) [36,37] was used to capture survivors’ physical activity level. The CUHK-PARCY grades the levels of physical activity according to the frequency, duration, and intensity (based on the metabolic equivalent of task (MET) score) of the activities. To help the participants understand the level of physical activity, examples of light (3 METs), moderate (5 METs) and vigorous (9 METs) activities were provided. The CUHK-PARCY has been previously validated among Chinese survivors of childhood cancer [34,36]. A lower score is indicative of poor physical activity. Scores from 0 to 3 represent low activity, scores from 4 to 6 represent moderate activity, and scores from 7 to 10 represent high activity [34,36].

(2) *Substance use.* Smoking, alcohol intake and substance abuse were self-reported using the adapted “Substance Use” scale of the Achenbach System of Empirically Based Assessment (ASEBA) Adult Self-report Checklist [38]. The survivors were asked about the average number of cigarettes they smoked per day, average number of days of heavy drinking, and average number of days they used drugs for non-medical purposes (including marijuana, cocaine or other psychoactive drugs) in the past 6 months. Drinking patterns were classified according to the Substance Abuse and Mental Health Services Administration (SAMHSA) criteria [39].

(3) *Inadequate sleep.* Survivors reported the average number of hours they slept every day (i.e., hours asleep) over the past month. According to the American Academy of Sleep Medicine and the Hong Kong Centre of Health Protection, fewer than 7 h of sleep at night is inadequate [40,41]. Subjective sleep-related fatigue level was assessed using the Sleep–Rest Fatigue subscale of the Pediatric Quality of Life Inventory Multidimensional Fatigue Scale (PedsQL-MFS) [42]. The PedsQL-MFS was used previously in a Chinese young-adult cancer population [34]. A score of ≤60 points represents a high level of sleep-related fatigue [43].

(4) *Long working hours.* Survivors who were employed reported their average number of working hours in the past month. Studies have reported that working ≥55 h per week *is associated with a higher risk of developing occupational health problems* [44]. As most people in Hong Kong work 6 days a week, working more than 9 h per day was considered detrimental to health.

### 2.6. Covariates

Demographic covariates (sex and age at follow-up), clinical information (cancer diagnosis, age at cancer diagnosis, and weight) and treatment variables (surgery, radiation and chemotherapy) were abstracted from the CMS.

The CHCs were from the Clinical Data Analysis and Reporting System (CDARS), which is a centralized clinical data repository for patients who received treatment from the public healthcare system of Hong Kong. CDARS documents the patients’ clinical diagnoses using International Classification of Diseases, Ninth Revision (ICD-9) codes and has previously been used in local pediatric oncology studies [45,46], and validated as a data source for epidemiological studies in Hong Kong [47]. We also screened for documented CHCs in the consultation notes of the CMS. In this study, CHCs referred to health conditions that persisted or developed two or more years after completion of therapy. CHCs of interest were limited to the cardiac, pulmonary, endocrine/metabolic and renal conditions, as these have been associated with neurocognitive function in sarcoma survivors [3,7,8]. The survivors also self-reported their highest educational attainment and employment status.

### 2.7. Statistical Analysis

Population characteristics and outcome measures were summarized for the overall cohort and stratified by diagnosis. The assumption of normality was tested using visual inspection (histograms and Q–Q plot) and the Kolmogorov–Smirnov test [48]. The survivors’ neurocognitive outcomes were compared with the normative data (t-score = 50) using one-sample *t*-test. Consistent with the approach adopted by other cognitive studies [32,34,49,50], the subsequent multivariable analyses only included neurocognitive measures that differed between the survivors and the normative samples at *p* < 0.05, after correcting for the false discovery rate (FDR) to avoid multiple comparisons [51]. For descriptive purposes, we estimated the prevalence of impairments within the study sample. Impairment was defined as a score worse than 1.5 standard deviations of the age-adjusted t-score, a definition which has been adopted by studies involving childhood cancer survivors in the literature [5,34,50].

General linear modeling (GLM) was conducted to identify factors associated with neurocognitive outcomes (*t*-scores as a continuous outcome variable) while adjusting for age at diagnosis, sex and highest educational attainment, which are well-established as covariates affecting neurocognitive outcomes in survivors of cancer [6,7,8]. The following risk factors investigated were determined *a priori* based on current literature on factors associated with cognitive outcomes in survivors of cancer: (1) CHCs (yes *versus* no) [3,7,8]; (2) clinical factors including cancer diagnosis (osteosarcoma *versus* STS) [5,8], history of relapse (yes *versus* no) [52], and weight status (normal weight *versus* overweight/obese) [27]; (3) treatment modality (surgery only *versus* surgery and chemotherapy *versus* surgery, chemotherapy and radiation) [3,7,8], and radiation sites (cranial radiation versus non-cranial radiation (other body sites) versus no radiation [7], and (4) treatment response (good (i.e., 27-week treatment) *versus* poor (i.e., 40-week treatment)). The associations were quantified using non-standardized point estimates (Est) and standard errors (SE).

The association between lifestyle factors and neurocognitive t scores was examined using GLM while adjusting for age at diagnosis, sex, education attainment and the above clinical covariates. Lifestyle factors that were significantly associated with neurocognitive outcomes were added to the GLM as interacting terms (i.e., presence of CHC × *factor*) to examine whether there were differences in neurocognitive outcomes among the subgroups of survivors with or without CHCs.

For all GLMs, visual graphical inspection of the diagnostic plots was used to examine the normality of residues and homogeneity of residual variance. Multicollinearity was tested by generating the variance inflation factor (VIF) for each independent variable, with a VIF higher than 5 indicating the presence of significant multicollinearity. All statistical analyses were performed using SAS (SAS 9.4, SAS Institute, Cary, NC, USA) and were two-tailed. The significance threshold was set at *p* < 0.05.

## 3. Results

The study population included 116 survivors (Table 1). Half of the survivors were male (51.7%), and their mean age at follow-up was 28.2 (SD = 8.2) years. They were diagnosed with osteosarcoma (49.1%) or STS (50.9%) at a mean age of 13.3 (SD = 7.2) years. Most of the survivors were treated with surgery (94.8%) and chemotherapy (81.9%). One third (31.0%) received radiation. At follow-up, 45% of them had developed at least one CHC. The most common CHCs were metabolic (23.3%), hearing (12.1%) and renal (10.3%) conditions. Slightly more than 23% were overweight or obese.

Neurocognitive performance of the study cohort fell below the published norms (mean = 50, SD = 10) on measures of visual memory (delayed recall) (*p* = 0.025), motor processing speed (*p* = 0.003), cognitive flexibility (*p* = 0.018) and multiple measures of attention, after adjusting for the false discovery rate (Table 2). A minority of the survivor population demonstrated impairment in attentiveness (4.3–13.0%), immediate recall (16.4%), visual memory (delayed recall) (19.0%), motor processing speed (34.5%) and cognitive flexibility (18.1%).

### 3.1. Associations between Clinical/Treatment Factors and Neurocognitive Outcomes

**Figure 1:**
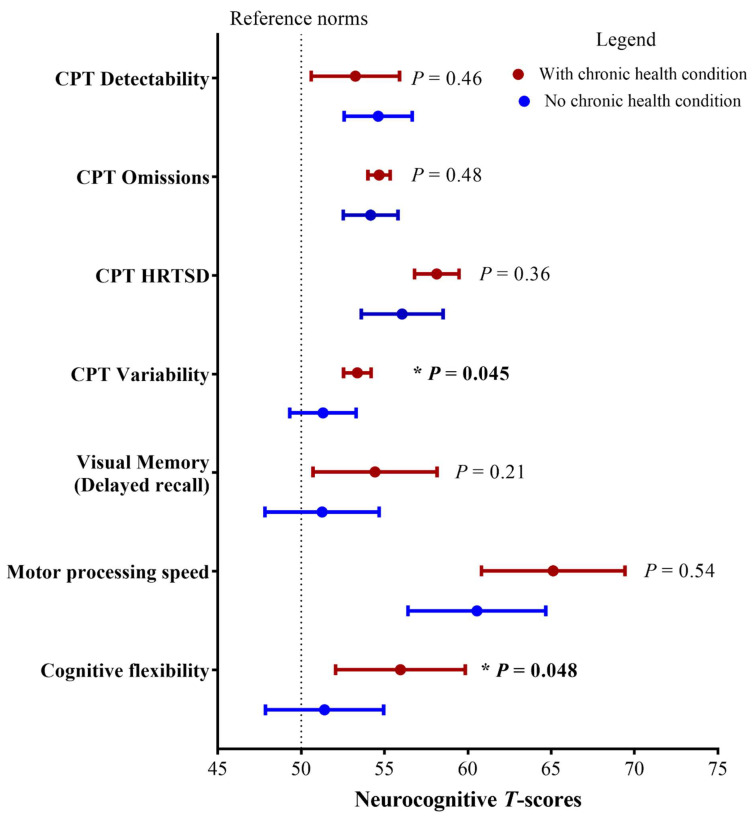
Association between chronic conditions and neurocognitive outcomes. CPT: Conners Performance Test-III for attention; HRTSD: hit reaction time standard deviation. There were 43 survivors who had developed cardiac, pulmonary, endocrine and metabolic or renal condition(s). These survivors’ neurocognitive scores were compared with survivors who did not develop chronic conditions using general linear modelling, adjusting for age at diagnosis, sex and highest educational attainment. A higher *T* score is indicative of worse functioning. * *p* < 0.05.

After adjusting for age at diagnosis, sex and educational attainment, the multivariate analyses showed that survivors who developed cardiopulmonary, endocrine, metabolic, or renal CHC (n = 43, 37.0%) demonstrated more performance impairments in attentiveness (*p* = 0.045) and cognitive flexibility (*p* = 0.048) than survivors who did not have CHC (Figure 1).

Survivors who were overweight/obese had more memory impairment than survivors with normal weight (*p* = 0.034) (Supplement Table S2). Among osteosarcoma survivors, poor treatment responders had poorer cognitive flexibility than good treatment responders (*p* = 0.043). Survivors who were treated with head/cranial radiation demonstrated worse attentiveness (CPT variability), as compared to survivors who did not receive any radiation (*p* = 0.031).

No significant association was identified between neurocognitive scores and cancer diagnosis in multivariable analyses (Supplement Table S2). The overall rates of impairment ranged from 1.8% to 29.8% for osteosarcoma survivors and 1.7% to 39.0% for STS survivors across the various neurocognitive domains (Supplement Table S3).

### 3.2. Lifestyle Factors

On a scale of 0 to 10, the overall median score for the CUHK-PARCY in the study population was 5 points (interquartile range (IQR): 2–6), which corresponds to moderate physical activity (Table 3). Slightly less than half of the survivors (43.1%) reported low physical activity, while 34.5% reported moderate physical activity and 22.4% reported high physical activity. More than one third (37.5%) of the survivors reported sleeping ≤7 h per day. The median sleep–rest fatigue score was 62.5 points (IQR: 50.0–70.8); more than half (58.6%) reported a high level of fatigue (score ≤ 60 points). Among employed survivors (n = 81), 18.5% reported working >9 h on an average working day.

Most of the survivors had not had alcohol (89.7%) in the past 6 months, whereas 12 (10.3%) identified themselves as drinkers. None of the drinkers were classified as “heavy drinkers” according to the SAMHSA criteria. Only seven survivors (6.0%) identified themselves as current smokers (range 2–20 cigarettes/day). Only one survivor reported using drugs for non-medical purposes.

### 3.3. Associations between Lifestyle Factors and Neurocognitive Outcomes

The multivariable analysis (Table 4) showed that physical inactivity was associated with inattentiveness, as reflected by worse scores on CPT detectability (Est = −0.88, SE = 0.28; *p* = 0.002) and CPT omissions (Est = −0.97, SE = 0.32; *p* = 0.003). Compared with individuals who never smoked, current smokers had poorer scores on CPT detectability (Est = 6.69, SE = 3.39; *p* = 0.048) and CPT omissions (Est = 8.96, SE = 3.81; *p* = 0.020). Survivors who consumed alcohol had a worse performance on CPT omissions (Est = 6.65, SE = 3.00; *p* = 0.029). Compared with survivors who reported working ≤ 9 h per day, survivors with longer working hours demonstrated more inattentiveness (*p*’s <0.05) and lower cognitive flexibility (Est = 5.22, SE = 1.80; *p* = 0.005). Sleep-related fatigue was associated with measures of inattentiveness (*p*’s < 0.05).

**Figure 2:**
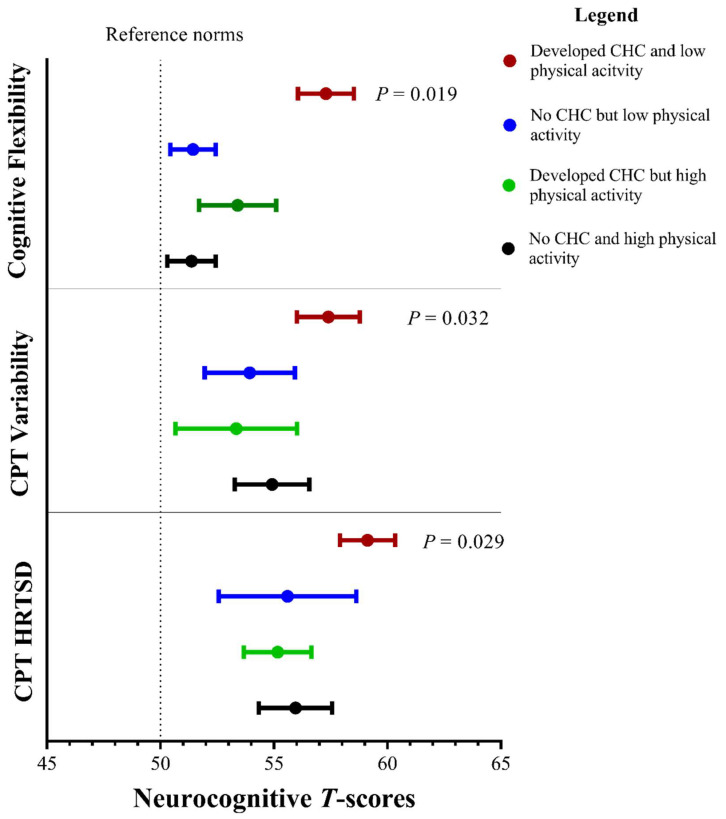
Interaction between chronic health conditions and physical activity. CHC: chronic health conditions; HRTSD: hit reaction time standard deviation. High physical activity refers to survivors who reported a score of ≥7 points on the CUHK-PARCY, which corresponds to the World Health Organization recommended ≥150 min of moderate-intensity aerobic physical activity per week. There was significant interaction for CHCs*physical activity for cognitive flexibility (*p* = 0.019), CPT variability (*p* = 0.032) and HRTSD (*p* = 0.029). This suggests that survivors who developed CHCs and reported low physical activity demonstrated the worst cognitive flexibility and attention outcomes, as compared to survivors in other subgroups.

Lifestyle factors that were significantly associated with neurocognitive scores were added to the GLM as interacting terms (presence of CHC × *factor*). Survivors who developed CHCs and reported poor physical activity demonstrated the worst attention and cognitive flexibility outcomes, as compared to the survivors in other subgroups (Figure 2). No statistically significant interactions between CHCs and other lifestyle variables were found.

## 4. Discussion

Up to one-third of AYA survivors of sarcoma demonstrated impairment in measures of inattention, motor processing speed and cognitive flexibility. Our results suggest associations between lifestyle factors and neurocognitive outcomes. Low physical activity was correlated with inattentiveness. Impairments were more pronounced among survivors who developed CHCs and were more physically inactive than their counterparts. Survivors who reported long working hours also demonstrated poorer attention and cognitive flexibility. These findings and emerging evidence from the existing literature [14], suggest that adverse functional outcomes in young survivors of cancer may be attributable to health behaviors. The promotion of positive health behaviors and lifestyle choices is imperative in high-risk subgroups of aging survivors with a poor health status.

The overall physical-activity level in the study cohort was low. Our data suggests that physical inactivity is associated with inattentiveness in young survivors of sarcoma. There is existing evidence supporting the positive neurophysiological effects of exercise in adult cancer populations [28,53], with emerging evidence showing a similar trend in younger cancer populations. Adult survivors of childhood cancer who report regular physical activity have fewer neurocognitive problems [27]. Exercise intolerance is associated with decreases in the performance of tasks involving focused attention, executive function, visual–motor processing speed and memory span in survivors of childhood ALL [16]. Our findings support existing evidence that exercise may confer neurocognitive benefits, in addition to physical health benefits, to AYA cancer survivors. Future health promotion efforts should include spreading awareness of the potential neurophysiological benefits of exercise and implementing exercise initiatives with the support of schools and community.

Our results show a novel finding on the interactions between physical inactivity and CHCs and their effects on attention and executive function. Around one third (37%) of the survivors developed clinical cardiopulmonary, metabolic and endocrine, or renal complications. Notably, our reported prevalence of CHCs was lower than previously published estimates which included systematic screening of late effects [5,7]. Survivors with CHCs who exercised the least frequently demonstrated the worst attention and cognitive flexibility outcomes. Higher order thinking abilities are regulated by the frontoparietal network and the ventral and dorsal attention networks of the brain, which are closely modulated by cerebral blood flow. It is plausible that exercise may increase the overall oxygen uptake efficiency of the brain [54] thereby improving cerebral perfusion and neural integrity in patients with CHCs [55]. One clinical implication of this finding is that although the development of CHCs in survivors may be irreversible, the promotion of physical activity in high-risk survivors may alter the trajectory of cognitive decline before the CHCs develop into functional impairment. Future work should include validating this finding in larger populations and developing exercise interventions targeted at cancer survivors with poor health status. Admittedly, it is a challenge to promote regular physical activity among this population, as a number of survivors with CHCs, prostheses or who have undergone radical surgery reported concerns with exercising *(personal communication)*. These survivors may benefit from the advice of exercise specialists who could counsel patients based on recommended exercise guidelines and provide recommendations on the appropriate type of aerobic and resistance training for sarcoma survivors. Emerging evidence supports the integration of an exercise specialist into the clinical team [56,57], though, unfortunately, this area is still at its nascent state in the context of Hong Kong. Future efforts should be targeted at providing practical support to facilitate the implementation of exercise guidelines and referrals to exercise specialists and supervised exercise programs.

Close to one fifth of the survivors reported working >9 h per day and one third slept fewer than 7 h per day. Globally, it has been reported that working long hours (≥55 h/week) is attributable to 3.7% and 6.9% of deaths from stroke and ischemic heart disease, respectively, with rates disproportionally higher in certain regions of Asia and the Western Pacific [44]. In our study, survivors who reported longer working hours demonstrated poorer attention and cognitive flexibility than those who reported shorter working hours. Although sleep hours were not associated with cognitive outcomes, sleep–rest fatigue was strongly correlated with inattentiveness. This finding should be interpreted cautiously due to the lack of a comprehensive measure of sleep (e.g., sleep quality, sleep latency and sleep efficiency) and information on occupation types and work schedule in this study population. Although we did not identify any interactions between CHCs and working hours or sleep that affect neurocognitive outcomes, it is plausible that survivors may be more susceptible to cognitive impairment and health effects of occupational risk factors [35]. In a similar vein, we previously reported that academic stress, another culturally relevant phenomenon in Asian societies, is associated with poorer neurobehavioral outcomes among school-aged survivors of childhood ALL in Hong Kong [34]. Future studies should include a more comprehensive assessment of culturally relevant occupational and academic stressors, and investigate how these potentially modifiable risk factors may affect functional outcomes in high-risk survivors.

The rates of smoking (6.0%) and alcohol consumption (10.3%) were low among AYA survivors of sarcoma. A national survey in Hong Kong reported that in 2019, among persons aged 15 to 45 years, only 8.8% drank alcoholic beverages regularly and 2.9% engaged in binge drinking in the 12 months preceding the survey [58]. Smoking prevalence in this age group was 9.5% in 2021 [59]. A smaller proportion of sarcoma survivors who were smokers demonstrated poorer attentiveness than survivors who never smoked. This is consistent with the known associations of nicotine dependence with attention deficits [60]. In addition, exposure to secondhand smoke is also associated with cognitive impairment [61]. This is concerning, as we have also previously reported that exposure to secondhand smoke in the home among local survivors of childhood cancer was surprisingly high (15%). This is probably because the smoking rates in individuals > 40 years of age (likely the age group of survivors’ parents or grandparents) remain relatively high in Hong Kong [15]. One US study reported that young adults with cancer history were more likely to report having ever used e-cigarettes than their peers without a cancer history [31], and vaping was associated with cognitive complaints in the general population [62]. Although the sale and manufacture of e-cigarettes has been illegal in Hong Kong since April 2022, continuous efforts should also be dedicated to discouraging survivors and their family members from such harmful practices.

Our study has a few limitations. First, the study sample size was small, as sarcomas have lower incidence and survival rates than more common cancers in pediatric and young-adult populations. Our study sample is heterogeneous in terms of demographics (age) and clinical characteristics (cancer diagnoses and age at cancer diagnosis). Since the primary aim was to explore the impact of lifestyle factors on neurocognitive outcomes, the associations that were identified in this study are still applicable to AYA survivors of this age spectrum. However, we also acknowledge that the lifestyle patterns of adolescent survivors, who were likely students, might be different from young-adult survivors who were working. We attempted to account for these baseline differences in our statistical models. Our results are also limited by the absence of a non-cancer control group. However, the impact of lifestyle factors on cognitive impairment has also been previously reported in the general population too [63,64]. As such, our association findings might still be valid and applicable to the emerging population of AYA cancer survivors globally. Second, we found that survivors who underwent cranial radiation and poor treatment responders (for osteosarcoma survivors) had worse attentiveness and cognitive flexibility, respectively. It is also reasonable to postulate that the high rates of motor-processing-speed impairment may be attributable to the persisting symptoms of numbness/tingling from vincristine-induced peripheral motor neuropathy. However, our institutional medical records did not comprehensively document cumulative doses of chemotherapy and dosimetry data. Third, the treatment protocols represented in this study cohort span across 1990s to 2000s. However, there have been no major changes in the treatment protocols for osteosarcoma in the past three decades [19,65]. The treatment modalities and chemotherapy drugs received by STS survivors in this study remain the backbone of conventional therapies for STS [66].

Although data on lifestyle factors were obtained through validated tools, these were still self-reports, which may be affected by biases in recall and social desirability. Further studies should adopt actigraphy measures to capture actual physical activity and sleep prospectively. Lastly, due to the constraint of space and time in a clinical setting, we could not include a more comprehensive assessment of other neurocognitive measures, such as global intelligence (verbal and non-verbal reasoning) and tests of academic achievement. As neurocognitive function is multifactorial in nature, there are other unmeasured social and environmental confounders that affect lifestyle factors and functional outcomes. This study was conducted during the COVID-19 pandemic, when the lifestyle factors of survivors may have been affected by city-wide infection control measures. A prospective follow-up study should evaluate the impact of culturally and regionally dependent factors on functional outcomes among survivors.

## 5. Conclusions

The current study captured attention, memory, processing-speed and cognitive-flexibility performance data using objective performance-based testing in a cohort of young Chinese survivors of osteosarcoma and STS, a relatively rare and understudied cancer population. Our findings suggest associations between health behaviors and neurocognitive function among young cancer survivors. This topic is of major public-health significance, as much emphasis is now placed on addressing the adverse health effects of the urban environment on the health outcomes of cancer survivors. Our results also suggest that physical inactivity is associated with worse attention outcomes, hence reinforcing the importance of existing initiatives to promote early exercise interventions to increase physical fitness among young cancer survivors. There is preliminary evidence showing that survivors with long working hours or inadequate sleep are more likely to have cognitive impairment than their counterparts, though this finding should be validated in larger studies with prospective assessment of occupational conditions and work-related stressors. Future work should investigate intervention targets and leverage health behaviors as modifiable risk factors to prevent neurocognitive dysfunction in this special population.

## Figures and Tables

**Table 1:** Characteristics of study population.

Characteristics	All Survivors N = 116	Osteosarcoma N = 57	Soft-Tissue Sarcoma N = 59
	n (%)	n (%)	n (%)
**Sex**			
Male	60 (51.7)	31 (54.4)	29 (49.2)
Female	56 (48.3)	26 (45.6)	30 (50.8)
Age at interview (years) *	28.2 (8.2)	29.3 (8.2)	27.2 (8.1)
>15–18 years	15 (12.9)	6 (10.5)	9 (15.3)
≥18–30 years	62 (53.4)	29 (50.9)	33 (55.9)
>30 years	39 (33.7)	22 (38.6)	17 (28.8)
Age of Diagnosis (years) *	13.3 (7.2)	13.0 (5.2)	13.5 (8.8)
<15 years (diagnosed at childhood)	82 (70.7)	44 (77.2)	38 (64.4)
≥15–18 years (diagnosed at adolescence)	19 (16.4)	8 (14.0)	11 (18.6)
≥18–39 years (diagnosed at young adulthood)	15 (12.9)	5 (8.8)	10 (17.0)
Years from diagnosis *	14.9 (7.6)	16.2 (7.5)	13.7 (7.5)
≤5 years	16 (13.8)	3 (5.3)	13 (22.0)
>5–10 years	21 (18.1)	11 (19.3)	10 (16.9)
>10–20 years	52 (44.8)	27 (47.4)	25 (42.3)
>20 years	27 (23.2)	16 (28.0)	11 (18.6)
**Education level**			
Secondary school or below	42 (36.2)	22 (38.6)	20 (33.9)
Post-secondary school or above	70 (60.3)	33 (57.9)	37 (62.7)
**Employment status**			
Employed	81 (69.8)	43 (75.4)	38 (64.4)
Not employed	20 (17.2)	8 (14.0)	12 (20.3)
Students	15 (12.9)	6 (10.5)	9 (15.3)
**Clinical characteristics**			
**Tumor site (for osteosarcoma)**			
Femur	-	32 (56.1)	
Tibia	-	14 (24.7)	
Humerus	-	5 (8.8)	
Fibula	-	3 (5.2)	
Others (hip, neck)	-	3 (5.2)	
**Tumor site (for soft-tissue sarcoma)**			
Lower extremity	-	-	18 (30.5)
Upper extremity	-	-	6 (10.2)
Abdomen/pelvis	-	-	17 (28.8)
Head and neck	-	-	10 (16.9)
Chest	-	-	8 (13.6)
**Subtypes (for soft-tissue sarcoma)**			
Rhabdomyosarcoma	-	-	15 (25.4)
Ewing sarcoma	-	-	11 (18.6)
Liposarcoma	-	-	6 (10.2)
Synovial Sarcoma	-	-	5 (8.5)
Clear Cell Sarcoma	-	-	4 (6.8)
PNET	-	-	3 (5.1)
Others	-	-	15 (25.4)
**Relapse**			
No	105 (90.5)	50 (87.7)	54 (91.5)
Yes	11 (9.5)	7 (12.3)	5 (8.5)
**CHCs**			
No	60 (51.7)	27 (47.4)	33 (55.9)
Yes	53 (45.7)	28 (49.1)	25 (42.4)
Endocrine #	8 (6.9)	1 (1.8)	7 (11.9)
Metabolic #	27 (23.3)	13 (22.8)	14 (23.7)
Renal #	12 (10.3)	12 (21.1)	0 (0)
Cardiovascular #	8 (6.9)	6 (10.5)	2 (3.4)
Pulmonary #	3 (2.6)	0 (0)	3 (5.1)
Other CHCs:			
Hearing	14 (12.1)	11 (19.3)	3 (5.1)
Behavioral	8 (6.9)	3 (5.3)	5 (8.5)
Second cancer	4 (3.4)	3 (5.3)	1 (1.7)
Musculoskeletal	4 (3.4)	1 (1.8)	3 (5.1)
Vision	4 (3.4)	0 (0)	4 (6.8)
Gastrointestinal	4 (3.4)	1 (1.8)	3 (5.1)
Neurological	5 (4.3)	0 (0)	5 (8.5)
Liver	3 (2.6)	1 (1.8)	2 (3.4)
Body mass index (years) *	21.7 (3.9)	21.6 (3.6)	21.7 (4.1)
Underweight	16 (13.8)	9 (15.8)	7 (11.9)
Normal	73 (62.9)	30 (52.6)	30 (50.8)
Overweight/obese	27 (23.3)	13 (22.8)	14 (23.7)
**Treatment characteristics**			
**Chemotherapy**			
No	21 (18.1)	5 (8.8)	16 (27.1)
Yes	95 (81.9)	52(91.2)	43 (72.9)
Doxorubicin	72 (62.1)	51 (89.5)	21 (35.6)
Cisplatin	51 (44.0)	49 (86.0)	2 (3.4)
High-dose methotrexate	50 (43.1)	49 (86.0)	1 (1.7)
Ifosamide	46 (39.7)	27 (47.4)	19 (32.2)
Etoposide	38 (32.8)	26 (45.6)	12 (20.3)
Vincristine	35 (30.2)	1 (1.8)	34 (57.6)
Actinomycin D	28 (24.1)	1 (1.8)	27 (45.8)
Cyclophosamide	22 (19.0)	3 (5.3)	19 (32.2)
Carboplain	6 (5.2)	4 (7.0)	2 (3.4)
Bleomycin	2 (1.7)	1 (1.8)	1 (1.7)
**Radiation**			
No	78 (67.2)	52 (91.2)	26 (44.1)
Yes	36 (31.0)	4 (7.0)	32 (54.2)
Cranial area	8 (6.9)	0 (0)	8 (13.6)
Non-cranial areas (other body sites)	28 (24.1)	4 (7.0)	24 (40.7)
**Surgery**			
No	5 (4.3)	0 (0)	5 (8.47)
Yes	110 (94.8)	57 (100)	53 (89.8)
**Treatment combinations**			
Surgery only	14 (12.1)	4 (7.0)	10 (16.9)
Surgery and chemotherapy	63 (54.3)	48 (84.2)	15 (25.4)
Surgery, chemotherapy and radiation	27 (23.3)	3 (5.3)	24 (40.7)
Others	12 (10.3)	2 (3.5)	10 16.9)

CHC: chronic health condition; N.A.: not applicable; PNET: primitive neuro-ectodermal tumor. * Presented as mean (standard deviation). # These CHCs are analyzed in subsequent multivariable analyses as predictors of neurocognitive function, based on evidence from the literature. The common examples of conditions for each CHC category are listed below: Endocrine: hypothyroidism, primary ovarian failure, male germ cell dysfunction; Metabolic: overweight/obesity, diabetes mellitus; Renal: chronic kidney dysfunction (e.g., Fanconi syndrome); Cardiovascular: hypertension, dyslipidemia, cardiomyopathy; Pulmonary: restrictive lung disease, abnormal pulmonary function

**Table 2:** Neurocognitive outcomes.

Neurocognitive Outcomes				
	Mean (SD) *T*-Scores *	Impaired% ^	95% CI ^	*p* ^†^
**Attention** *(* *Conners Continuous Performance Test-III)*				
Inattentiveness:				
Omission	54.4 (4.9)	4.3	0–8.0	**0.0003**
Detectability	54.0 (8.6)	13.0	5.4–16.9	**0.0003**
Variability	55.3 (6.9)	4.3	0–8.0	**0.0003**
HRT SD	57.2 (7.0)	9.4	4.1–14.8	**0.0003**
Impulsivity:				
Perseverations	52.4 (5.4)	0.8	0–2.5	1.00
Commission	50.6 (9.8)	0.8	0–2.5	0.51
Vigilance:				
HRT ISI change	51.2 (7.6)	1.7	0–4.1	0.13
Sustained attention:				
HRT block change	49.3 (8.5)	0.8	0–2.5	0.47
**Memory** *(Modified Taylor Complex Figure)*				
Visual memory (Immediate recall)	52.6 (13.7)	16.4	9.6–23.1	0.067
Visual memory (Delayed recall)	53.1 (13.4)	19.0	11.8–26.1	**0.025**
**Motor processing speed**				
Visual search *(TMT-A)*	48.7 (9.4)	6.0	1.7–10.4	0.17
Motor processing speed *(GPB)*	62.3 (17.2)	34.5	25.8–43.1	**0.003**
**Cognitive flexibility** *(TMT-B)*	53.5 (13.9)	18.1	11.1–25.1	**0.018**

CI: confidence interval; GPB: grooved pegboard; HRT: hit reaction time; ISI: inter-stimulus intervals; SD: standard deviation; TMT: trail making test. * All neurocognitive measures were transformed into age-adjusted *t* scores (mean = 50; standard deviation = 10) using published reference norms (Supplement Table S1). A higher score is indicative of worse functioning. ^ To estimate the prevalence of impairments within the study sample, impairment was defined as ≥1.5 standard deviation of age-adjusted *T*-scores of reference norms. The impairment rates and 95% CIs were presented. As a reference, the expected rates of global impairment for individuals aged 15 to 39 is between 4% and 6%. ^†^ Comparison with the reference norms was conducted using one-sample *t*-test. Only measures that were significantly different from reference norms at *p* < 0.05 (adjusting for false discovery rate) were included in subsequent analyses for examining associations with risk factors.

**Table 3:** Lifestyle factors.

Lifestyle Factors	Number (%)
Physical activity * median (IQR) CUHK-PARCY score	5 (2–6) points
Low level (0 to 3 points)	50 (43.1%)
Moderate level (4 to 6 points)	40 (34.5%)
High level (7 to 10 points)	26 (22.4%)
**Sleep**	
Self-reported average hours of actual sleep †	
<7 h/day	42 (37.5%)
≥7 h/day	70 (62.5%)
Median (IQR) sleep–rest fatigue score ^	62.5 (50.0–70.8)
Low level of fatigue	48 (41.4%)
High level of fatigue	68 (58.6%)
**Working hours ^†^**	
Self-reported average working hours/day	
≤9 h/day	66 (81.5%)
>9 h/day	15 (18.5%)
**Alcohol consumption**	
Non-drinker	104 (89.7%)
Drinkers ^Ɨ^	12 (10.3%)
*No. of heavy drinking days during the past 6 months (range)*	(1–20)
**Smoking**	
Never smokers	109 (94.0%)
Current smoker	7 (6.0%)
*Average no. of cigarettes/day during the past 6 months (range)*	(2–20)
**Substance abuse**	
No	115 (99.1%)
Yes	1 (0.9%)

* According to the CUHK-PARCY, a score of 7 or above fulfils the World Health Organization recommendation of 150 min of moderate-intensity aerobic physical activity per week. † Invalid response (n = 3) ^ A lower score represents a higher level of fatigue. A score of ≤60 points is indicative of high level of fatigue. † For working or employed survivors only. Ɨ None of the survivors were classified as “heavy alcohol use”, which is defined as heavy drinking on average 5 or more days in the past month by the Substance Abuse and Mental Health Services Administration.

**Table 4:** Association between lifestyle factors and neurocognitive outcomes.

	CPT Detectability *			CPT Omissions *			CPT HRTSD *			CPT Variability *			Visual Memory (Delayed Recall) *			Motor Processing Speed *			Cognitive Flexibility *		
	Est	SE	*p*	Est	SE	*p*	Est	SE	*p*	Est	SE	*p*	Est	SE	*p*	Est	SE	*p*	Est	SE	*p*
**Physical activity ^**	−0.88	0.28	**0.002**	−0.97	0.32	**0.003**	−0.18	0.23	0.44	−0.39	0.22	0.084	−0.66	0.45	0.14	−0.13	0.56	0.82	−0.61	0.46	0.19
**Smoking**																					
Current/ever	6.69	3.39	**0.048**	8.96	3.81	**0.020**	−0.71	2.77	0.79	−0.79	2.71	0.77	7.44	5.29	0.16	−6.55	6.41	0.31	−4.08	5.52	0.46
Never	Ref			Ref			Ref			Ref			Ref			Ref			Ref		
**Alcohol**																					
Drinkers	4.12	2.69	0.13	6.65	3.00	**0.029**	0.40	2.19	0.85	1.82	2.13	0.39	−0.92	4.36	0.83	9.98	5.18	0.056	5.76	4.33	0.18
Non-drinkers	Ref			Ref			Ref			Ref			Ref			Ref			Ref		
**Sleep**																					
Sleep hours																					
<7 h/day	1.90	1.69	0.26	3.24	1.92	0.095	0.44	1.40	0.75	0.75	1.36	0.58	4.30	2.62	0.10	2.00	2.98	0.50	1.33	2.78	0.63
≥7 h/day	Ref			Ref			Ref			Ref			Ref			Ref			Ref		
Sleep–rest fatigue ^†^	−0.07	0.04	0.08	−0.05	0.04	0.28	−0.06	0.03	**0.041**	−0.08	0.03	**0.005**	−0.03	0.06	0.64	0.03	0.08	0.70	0.01	0.06	0.84
**Working hours ^Ɨ^**																					
>9 h/day	5.42	2.33	**0.023**	5.94	2.61	**0.025**	2.88	2.24	0.20	2.74	2.16	0.21	0.13	3.95	0.97	6.27	4.62	0.17	5.22	1.80	**0.005**
≤9 h/day	Ref			Ref			Ref			Ref			Ref			Ref			Ref		

CPT: Conners performance Test-III for attention; Est: estimate; HRTSD: hit reaction time standard deviation; Ref: reference group; SE: standard error. * A higher score was indicative of worse functioning. All statistical models were adjusted for age at diagnosis, sex, educational attainment and clinical/treatment variables in Supplement Table S2. ^ A lower score represents lower physical activity.† A lower score represents higher level of sleep-rest fatigue. ^Ɨ^ For working or employed survivors only.

## Data Availability

The data presented in this study are available on request from the corresponding author. The data are not publicly available due to due to patient privacy concerns. A portion of the results will be presented at the International Cognition and Cancer Taskforce (ICCTF) meeting in 30–31 January 2023, San Diego, USA.

## Supplementary Materials

The following supporting information can be downloaded at: https://www.mdpi.com/article/10.3390/cancers15030799/s1, Table S1 Neurocognitive Measures and Sources of Reference Norms; Table S2 Association between Clinical/Treatment Factors and Neurocognitive Outcomes; Table S3 Neurocognitive Outcomes Stratified by Cancer Diagnosis.
