# Supplementary material for: Associations between Lifestyle Factors and Neurocognitive Impairment among Chinese Adolescent and Young Adult (AYA) Survivors of Sarcoma
Source: Cancers (Basel). 2023 Jan 28;15(3):799. doi: 10.3390/cancers15030799 (PMC9913447; doi:10.3390/cancers15030799)
Supplement: Supplementary file 1 [file cancers-15-00799-s001.zip › cancers-2115361-supplementary.pdf]

Associations between Lifestyle Factors and Neurocognitive Impairment among Chinese Adolescent and Young Adult (AYA) Survivors of Sarcoma

Yin Ting Cheung, Chung Tin Ma, Michael Can Heng Li, Keary Rui Zhou, Herbert Ho Fung Loong, Agnes Sui Yin Chan, Kwok Chuen Wong, Chi Kong Li

Table S1. Neurocognitive Measures and Sources of Reference Norms.

Neurocognitive Outcomes	Measures and Domains	Reference Norms Data
**Attention**	*Inattentiveness:*<br>CPT omissions<br>CPT detectability<br>CPT variability<br>CPT hit reaction time standard deviation<br><br>*Impulsivity:*<br>CPT commissions<br>CPT perseverations<br><br>*Sustained attention:*<br>HRT block change<br><br>*Vigilance:*<br>HRT ISI change	Published norms [21]
**Memory**	Modified Taylor Complex Figure [22]	Age- and sex-matched norms [22]
**Motor processing speed**	*Visual search:*<br>Trail Making A [23]	Age-matched Chinese norms[24,25]
	Motor processing speed:<br>Grooved Pegboard [23,26]	Age- and sex-matched norms [26]
**Cognitive flexibility**	*Cognitive flexibility:*<br>Trail Making Test B [23]	Age-matched Chinese norms [24,25]

CPT-III: Conners Continuous Performance Test (3rd Edition)

Table S2. Association between Clinical/Treatment Factors and Neurocognitive Outcomes.

	CPT Detectability*			CPT Omissions*			CPT HRTSD*			CPT Variability*			Visual memory (Delayed Recall)*			Motor processing speed*			Cognitive flexibility*		
	Est	SE	*P*	Est	SE	*P*	Est	SE	*P*	Est	SE	*P*	Est	SE	*P*	Est	SE	*P*	Est	SE	*P*
**Base model**																					
**Sex**																					
Female	0.84	1.62	0.60	-0.79	1.83	0.66	1.67	1.30	0.20	2.16	1.27	0.093	2.47	2.51	0.32	11.1	3.1	**0.004**	-2.46	2.58	0.35
Male	Ref			Ref			Ref			Ref			Ref			Ref			Ref		
**Education attainment**																					
Secondary school or below	2.42	1.71	0.16	2.14	1.96	0.27	0.06	1.42	0.97	2.31	1.37	0.095	4.26	2.66	0.11	2.42	3.02	0.42	6.06	2.76	**0.030**
Post-secondary school or above	Ref			Ref			Ref			Ref			Ref			Ref			Ref		
**Age at diagnosis ^**	-0.02	0.11	0.87	-0.04	0.12	0.74	-0.13	0.09	0.15	-0.09	0.09	0.28	0.12	0.17	0.48	-0.40	0.21	0.061	-0.13	0.18	0.49
**Clinical variables**																					
**Diagnosis ^**																					
STS	-0.09	1.63	0.95	-0.59	1.84	0.74	0.77	1.31	0.56	-1.18	1.27	0.35	-2.57	2.52	0.31	-1.68	3.07	0.58	-1.45	2.61	0.58
Osteosarcoma	Ref			Ref			Ref			Ref			Ref			Ref			Ref		
Relapse ^																					
Yes	3.78	2.66	0.15	1.31	1.49	0.38	1.36	2.16	0.52	1.48	2.10	0.48	4.39	4.14	0.29	0.10	5.03	0.98	8.33	4.24	0.052
No	Ref			Ref			Ref			Ref			Ref			Ref			Ref		
**Weight status ^**																					
Overweight/ obese	0.86	1.93	0.87	1.71	2.18	0.43	0.62	1.56	0.69	-0.79	1.52	0.60	5.72	2.56	**0.034**	-0.42	3.63	0.91	1.45	3.10	0.64
Normal/ underweight	Ref			Ref			Ref			Ref			Ref			Ref			Ref		
**Treatment variables**																					

**Treatment ^**																					
Surgery, chemotherapy & radiation	-2.87	3.19	0.37	-5.73	3.56	0.11	2.65	2.59	0.31	2.65	2.59	0.31	11.04	5.38	**0.043**	2.09	6.03	0.72	-3.00	5.15	0.56
Surgery & chemotherapy	-4.52	3.71	0.22	-6.88	4.14	0.10	2.29	3.02	0.44	2.29	3.02	0.44	4.49	4.63	0.33	3.57	7.18	0.62	-6.16	5.99	0.31
Surgery only	Ref			Ref			Ref			Ref			Ref			Ref			Ref		
**Radiation**																					
Cranial radiation	0.88	3.38	0.79	0.36	3.84	0.92	4.22	2.70	0.12	5.71	2.61	**0.031**	7.16	5.22	0.17	0.33	6.33	0.95	7.84	5.31	0.14
Radiation (other body sites)	-1.72	1.94	0.37	0.88	2.21	0.68	-1.29	1.55	0.40	-0.40	1.50	0.78	2.70	3.00	0.37	-0.47	3.75	0.90	-4.89	3.06	0.11
No radiation	Ref			Ref			Ref			Ref			Ref			Ref			Ref		
**Treatment duration ^ †**																					
40 weeks (poor responder)	0.76	2.75	0.78	1.47	2.97	0.62	2.70	1.98	0.18	1.48	1.34	0.27	1.22	3.86	0.75	8.92	6.04	0.14	5.96	2.31	**0.043**
27 weeks (good responder)	Ref			Ref			Ref			Ref			Ref			Ref			Ref		

CPT: Conners performance Test-III for attention; Est: estimate; HRTSD: hit reaction time standard deviation; Ref: reference group; SE: standard error

* A higher value was indicative of worse functioning.

^ Association between each clinical or treatment variable and neurocognitive outcome was tested using general linear models, adjusted for age at diagnosis, sex and highest education attainment.

† Among osteosarcoma survivors only

Table S3. Neurocognitive Outcomes Stratified by Cancer Diagnosis.

Neurocognitive outcomes								
	**Mean (SD)** *T*-Scores*	**Impaired %^**	**95% CI^**	***P*** †	**Mean (SD)** *T*-Scores*	**Impaired %^**	**95% CI^**	***P*** †
	**Osteosarcoma**				**Soft-tissue sarcoma**			
**Attention** *(Conners Performance Test-II)*								
Omission (inattentiveness)	54.0 (6.3)	1.8	0 – 5.2	**0.0003**	54.8 (2.6)	1.7	0 – 5.0	**0.0003**
Detectability (inattentiveness)	54.0 (8.8)	14.0	5.0 – 23.1	**0.0024**	53.8 (8.4)	8.4	1.4 – 15.6	**0.0020**
Variability (inattentiveness)	55.9 (4.6)	21.0	10.5 – 31.6	**0.0003**	54.7 (8.5)	20.3	10.1 – 30.7	**0.0003**
HRT SD (inattentiveness)	56.8 (6.4)	8.8	1.4 – 16.1	**0.0003**	57.5 (7.6)	10.1	2.5 – 17.9	**0.0003**
Perseverations (impulsivity)	53.0 (3.3)	1.8	0 – 5.2	**0.0003**	51.8 (6.8)	0	0 – 0	0.066
Commission (impulsivity)	50.9 (10.1)	1.8	0 – 5.2	0.54	50.3 (9.4)	0	0 – 0	0.81
HRT ISI change (vigilance)	49.8 (7.9)	1.8	0 – 5.2	0.89	52.5 (7.1)	3.4	0 – 8.0	**0.019**
HRT block change (sustained attention)	47.9 (6.5)	1.8	0 – 5.2	**0.037**	50.7 (9.9)	1.7	0 – 5.0	0.69
**Memory** *(Modified Taylor Complex Figure)*								
Verbal memory (Immediate recall)	52.0 (13.6)	17.5	7.7 – 27.4	0.35	53.2 (13.9)	15.3	6.0 – 24.4	0.10
Verbal memory **(**Delayed recall)	51.7 (13.4)	17.5	7.7 – 27.4	0.40	54.5 (13.4)	20.3	10.0 – 30.6	**0.021**
**Motor processing speed**								
Visual search *(TMT-A)*	47.6 (7.0)	3.5	0 – 8.3	0.12	49.8 (11.3)	8.5	1.3 – 15.6	0.87
Motor processing speed *(GPB)*	61.2 (18.3)	29.8	18.0 – 41.7	**0.0003**	63.5 (16.2)	39.0	26.5 – 51.4	**0.0003**
**Cognitive flexibility** *(TMT-B)*	52.8 (13.7)	21.1	10.5 – 31.6	0.17	54.7 (14.2)	15.3	6.1 – 24.4	**0.0003**

CI: confidence interval; GPB: Grooved Pegboard; HRT: hit reaction time; ISI: inter-stimulus Intervals; SD: standard deviation; TMT: Trail Making Test

* All neurocognitive measures were transformed into age-adjusted *T*-scores (mean=50; standard deviation=10) using published reference norms (Supplement 1). A higher score is indicative of worse functioning.

^ To estimate the prevalence of impairments within the study sample, impairment was defined as ≥1.5 standard deviation of sex- and age-adjusted *T*-scores of reference norms. The impairment rates and 95% CIs were presented.

† Comparison with the reference norms was conducted using one-sample t-test.
